# Simulating the physician as healthcare manager: An innovative course to train for the manager role

**DOI:** 10.3205/zma001040

**Published:** 2016-05-17

**Authors:** Maximilian Gradel, Stefan Moder, Leo Nicolai, Tanja Pander, Boj Hoppe, Severin Pinilla, Philip Von der Borch, Martin R. Fischer, Konstantinos Dimitriadis

**Affiliations:** 1 LMU Munich, Institut für Didaktik und Ausbildungsforschung in der Medizin, Munich, Germany; 2 Universityhospital Munich, Medzinische Klinik und Poliklinik IV, Munich, Germany; 3 LMU Munich, Neurologische Klinik und Poliklinik, Munich, Germany

**Keywords:** manager role, physician as manager, healthcare management, medical curriculum, simulation, gaming simulation

## Abstract

**Introduction: **During their formal studies medical students acquire extensive medical expertise. However, the medical profession demands additional competencies, such as those involved in efficient resource allocation, business administration, development, organization, and process management in the healthcare system. At present students are not sufficiently prepared for the physician’s role as manager. In response, we designed the seminar course, MeCuM-SiGma, to impart basic knowledge about healthcare policy and management to students of medicine. This project report describes our teaching strategies and the initial evaluation of this educational project.

**Project description: **In this semester-long, seminar course introduced in 2010, medical students gather experience with the competencies mentioned above as well as learn basic management skills. The course is offered each winter semester, and students sign up to attend voluntarily; course coordination and organization is done on a voluntary basis by physicians and employees of the Mentoring Office (MeCuM-Mentor) at the Medical School of the Ludwig Maximilian University (LMU) in Munich, Germany. The course is open to all students enrolled at the two medical schools in Munich.

During the first part of this elective, students learn about the basic principles of the German political and healthcare systems in case-based, problem-based tutorials led by trained tutors and in lectures held by experts.

In the second part of the course students take on the roles of the University Hospital’s executive board of directors and supervisory board to work on an existing hospital project as a group within the scope of a simulation. This phase of the course is accompanied by workshops conducted in cooperation with university-based and off-campus partners that address the procedural learning objectives (teamwork, project management, negotiation strategies, etc.).

A suitable, authentic issue currently facing the hospital is selected in advance by the course organizers in coordination with the hospital’s executive board. Students then work on this issue in the third and final phase of the course under the supervision of tutors and with assistance from hospital employees. At the end of the course the students formally present the results of their work to the hospital’s executive and supervisory boards.

**Results: **The course undergoes written student evaluation, a round of oral feedback, evaluation of the final projects, and feedback from the hospital’s executive and supervisory boards. All attendees to date have reported a substantial gain in general knowledge and increased knowledge about the healthcare system, and rate the relevance of the course as being high. The majority felt the content was important for their future practice of medicine. Overall, students evaluated the course very positively [overall rating on a six-point grading scale (1=excellent; 6=unsatisfactory): 1.28 (mean)±0.45 (standard deviation)].

**Discussion: **The importance of the physician’s role as manager in medical organizations and as a guiding force in the healthcare system is neglected in medical degree programs. Our seminar course attempts to address this shortcoming, is the object of great interest and receives positive evaluations from seminar participants, our cooperative partners and the executive and supervisory boards of the University Hospital in Munich.

## Zusammenfassung

**Einleitung:** Medizinstudierende erlangen durch ihre Ausbildung ein umfassendes medizinisches Fachwissen. Allerdings erfordert der Arztberuf zusätzliche Fähigkeiten wie Ressourcenallokation, Wirtschaftlichkeit, Konzeption, Organisation und Prozessmanagement im Gesundheitssystem. Auf die Rolle des Arztes als Manager werden die Studierenden nicht ausreichend vorbereitet. Deshalb entwickelten wir den Kurs MeCuM-SiGma, welcher Medizinstudierenden die Grundlagen von Gesundheitspolitik und -management vermittelt. In diesem Projektbericht stellen wir das didaktische Konzept und erste Ergebnisse der Evaluation des Lehrprojekts vor.

**Projektbeschreibung: **Der seit 2010 bestehende, einsemestrige Kurs vermittelt Medizinstudierenden neben den oben bereits erwähnten Kenntnissen grundlegende Managementkompetenzen. Das immer im Wintersemester stattfindende, freiwillige Lehrprojekt wird ehrenamtlich von Ärzten und Mitarbeitern des Mentoring-Büros (MeCuM-Mentor) der medizinischen Fakultät der LMU München koordiniert sowie organisiert und steht allen Medizinstudierenden der beiden medizinischen Fakultäten in München zur Teilnahme offen.

In der ersten Phase dieses Zusatzangebots lernen die Studierenden in fallbasierten, problem-orientierten Tutorials unter Anleitung von geschulten Tutoren und in Expertenvorträgen u.a. die Grundlagen des politischen Systems und des Gesundheitssystems in Deutschland kennen.

In der zweiten Phase nehmen die Teilnehmer die Rollen der Mitglieder des Vorstandes sowie des Aufsichtsrates des Klinikums der Universität München ein und bearbeiten in Teamarbeit im Rahmen einer Simulation ein bereits bestehendes Projekt des Klinikums. Begleitet wird diese Phase von Workshops zu den prozeduralen Lernzielen (u.a. zu Teamarbeit, Projektmanagement und Verhandlungsstrategien), die in Kooperation mit inner- und außeruniversitären Kooperationspartnern durchgeführt werden.

In enger Zusammenarbeit mit dem Vorstand des Klinikums wird von den Kursverantwortlichen im Vorfeld eine geeignete reale Problemstellung ausgewählt, mit der sich der Klinikumsvorstand aktuell befasst. Dieses bearbeiten die Studierenden in der dritten und letzten Phase unseres Kurses unter Supervision von Tutoren und unter Mithilfe von Klinikumsmitarbeitern. Zum Abschluss stellen die Teilnehmer ihre Ergebnisse dem realen Vorstand und Aufsichtsrat in einer Abschlusspräsentation vor.

**Ergebnisse: **Der Kurs wird mittels einer schriftlichen Evaluation der Teilnehmer, einer mündlichen Feedbackrunde, einer Evaluation der Abschlussprojekte und eines Feedbacks von Vorstand und Aufsichtsrat des Klinikums evaluiert. Alle bisherigen Teilnehmer maßen dem Kurs einen großen inhaltlichen Informationsgewinn zu, bekundeten einen Zuwachs an Wissen zum Gesundheitswesen und schätzten die Relevanz der Kursziele hoch ein. Die Mehrheit fand die Inhalte wichtig für ihre spätere ärztliche Tätigkeit. Insgesamt wurde der Kurs von den Teilnehmern sehr gut [Gesamtnote auf sechsstufiger Notenskala (1=sehr gut; 6=ungenügend): 1,28 (Mittelwert)±0,45 (Standardabweichung)] bewertet.

**Diskussion: **Die Bedeutung der Rolle des Arztes als Manager sowohl in medizinischen Organisationeinheiten als auch als Weichensteller im Gesundheitssystem wird in der medizinischen Ausbildung vernachlässigt. Unser Kursangebot beginnt diese Lücke zu schließen und wird mit großem Interesse und hervorragender Evaluation von Teilnehmern, Kooperationspartnern, Vorstand und Aufsichtsrat unserer Universitätsklinik angenommen.

## Introduction

Technological progress, biomedical innovations, the demographic shift and epidemiological transition are often cited as the greatest challenges to the healthcare system [1], [2], [3]. The German healthcare system is also facing a financial deficit mainly for these reasons [4]. The hospital sector appears to be most affected by current reforms and restructuring.

According to a study published by the German Hospital Institute (Deutsche Krankenhausinstitut), over 40% of hospitals have an annual deficit [5]. This economic pressure on hospital management falls heavily on those employees who are responsible for generating profit: the physicians whose tasks go beyond treating patients to include additional skills and abilities such as resource allocation, development, organization and process management, not only when managing a hospital ward or private practice but also in key leadership positions within the healthcare system [6]. Understanding the processes within the healthcare system and how to handle resources responsibly and sustainably form an important basis for medical practice. In the Anglo-American countries the role of the physician as healthcare manager has already been included in a variety of competency-based catalogues (CanMEDs, ACGME Competency-based Residency Education, AoMRC and the NHS Institute for Innovation and Improvement Medical Leadership Competency Framework) [7], [8], [9]. Looking ahead to the future, the German National Competency-based Catalogue of Learning Objectives in Undergraduate Medical Education (NKLM) is also trying to do justice to these roles [http://www.nklm.de/].

Although the currently valid medical licensure regulations (Approbationsordnung) do require academic credits in the three combined interdisciplinary topics of medical economics, healthcare systems, and public health, in most national and international curricula this particular aspect of medical education decidedly takes a backseat to the role of “medical expert” [http://www.gesetze-im-internet.de/_appro_2002/BJNR240500002.html]. On a survey in the United States less than half of the medical students questioned indicated that they were satisfied with their education regarding the practice of medicine (medical economics, health care systems, managed care, practice management, medical record keeping) [10]. Similar results were seen for young assistant physicians in the Netherlands, Denmark and Australia [11]. An extensive overview of existing programs in North America, Europe and Asia identified a major need for training in regard to these skills [12]. In Germany, a longitudinal study on the development and self-perception of management skills, as they are described in the CanMEDs roles, showed that medical students possess these only at a very rudimentary level [13]. 

To meet this need, the seminar, **Me**dizinisches **Cu**rriculum **M**ünchen - **Si**mulation **G**esundheits**ma**nagement (MeCuM-SiGma), was designed and implemented in 2010 by assistant physicians at the Munich University Hospital (KUM) and, to a significant extent, by students enrolled in different departments and schools (Medicine, Political Science, Business Administration, Economics) of the Ludwig Maximilian University (LMU). Today, students are still closely involved not only in the organization and coordination, but also in determining the focus of the educational project.

## Project description

MeCuM-SiGma is open to all medical students enrolled at either medical school in Munich (LMU or TUM) as an elective course. Interested students apply for acceptance to attend the seminar by submitting a letter of intent and an essay on an issue pertaining to healthcare policy. The applications are anonymized and sent to the members of a committee comprised of course organizers and former participants who then individually evaluate the statements and essays based on a set of defined criteria. The committee then convenes to discuss, evaluate and select 24 candidates for acceptance to the seminar; on average between 25 and 30 applications are submitted. A website has been created for the course [http://www.mecum-sigma.de] where students can find information and submit applications. The restricted-access area of the site provides seminar participants with current course materials, meeting dates and times, as well as an exportable calendar. In addition, the seminar organizers host a closed group on FaceBook enabling vertical exchange between current course participants and alumni.

### Learning objectives

MeCuM-SiGma aims to prepare physicians-to-be for the social responsibility they will carry, beyond their role as medical experts, as stakeholders in the healthcare system. The seminar’s learning objectives focus on the role of the physician as manager to place future physicians in a position to “participate in activities that contribute to the effectiveness of their healthcare organizations and systems.” They should be able to “manage their practice and career effectively” and “allocate finite healthcare resources appropriately.” And they should, when needed, be able to “serve in administration and leadership roles” ([7], p. 6).

Yet, the learning objectives of MeCuM-SiGma do not limit the role of physician as manager to the definitions provided by the competency-based catalogues, but also place it within the context of management theory. Accordingly, management skill is the ability to successfully fulfill management functions such as planning, organization, coordination and control [14], [15], [16], [17]. These management skills also have significance for the practice of medicine at any level of a given hierarchy. The learning objectives are presented in detail in table 1 (Tab. 1).

#### Course sequence

**Figure 1:**
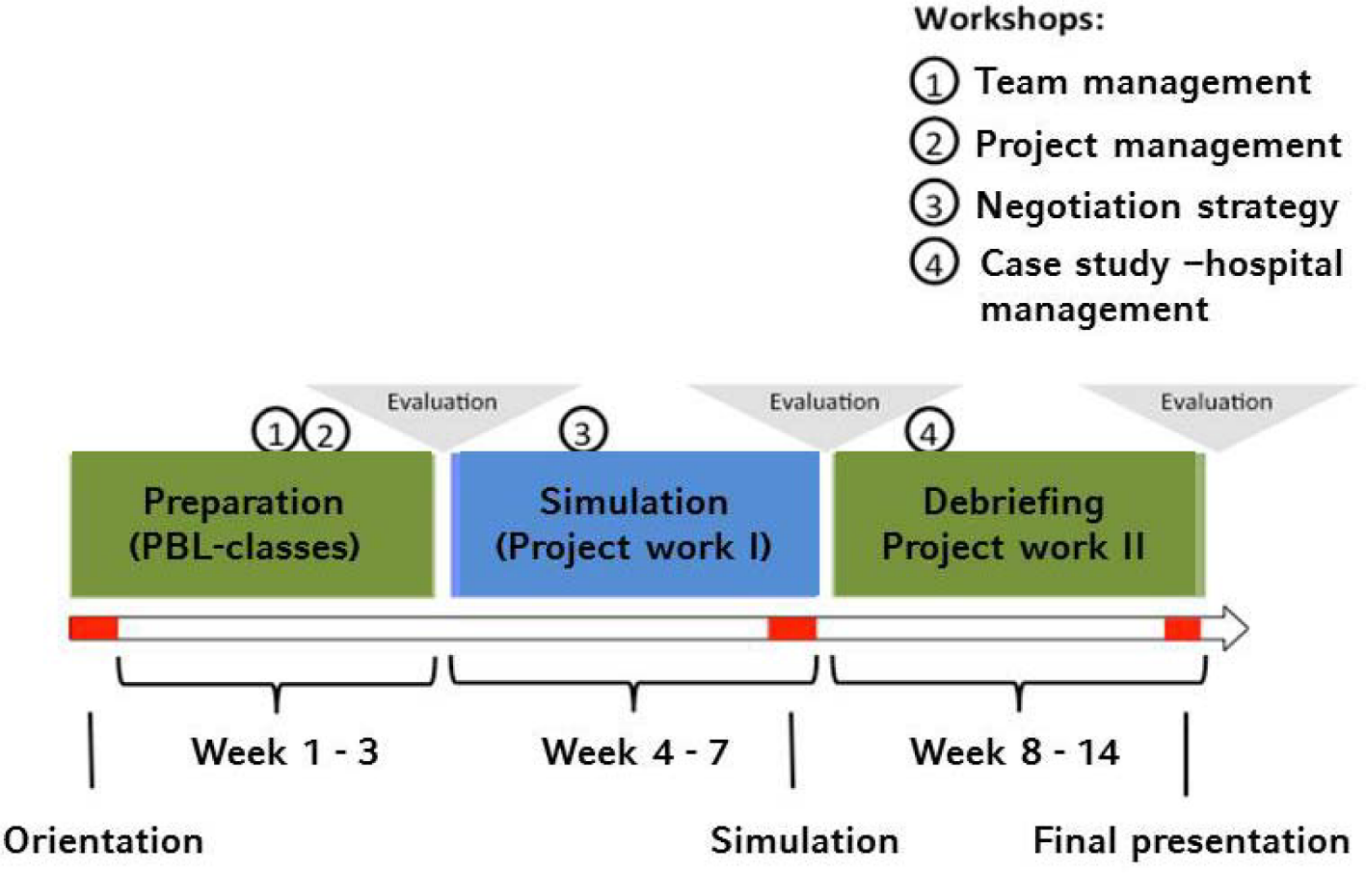
Course structure

The seminar takes place over the course of one semester and is divided into three sequential phases that build off the preceding ones in terms of content and organization (figure 1). The complex course content is taught using several teaching methods, with gaming simulation and an individual project serving as the basis. According to Cecchini, gaming simulation can be classified using a three-dimensional model. The three levels encompass the simulation, the role, and the game. The more balanced the three levels are (normal form), the more the participants find it to be realistic and the more efficient the learning atmosphere is [18]. As described in the first phase, this seminar aims for a normal form. In terms of organizing course content, the gaming simulation is preceded and followed by traditional teaching methods [19].

Making use of problem-based learning tutorials and an interactive lecture, the first phase serves as preparation and motivation, as well as an opportunity to activate prior knowledge.

Simulated gaming then follows in the second phase, while at the beginning of the third phase debriefing takes place during which the relevant learning content and objectives are systematically emphasized and expanded based on the learning experiences of the seminar participants. This enables immediate application of the acquired knowledge to the subsequent seminar project (see table 2 (Tab. 2)). The two-hour seminar sessions are held on Mondays; additional meetings primarily include all-day workshops, the simulation and final presentation (see figure 1 (Fig. 1)).

#### Phase I: Lectures and problem-based learning tutorials: The German political system, healthcare system and healthcare management

The course begins with an interactive survey presented by the instructors, the purpose of which is to cover and impart basic knowledge regarding the healthcare system and bring all the participants up to the same level. In addition, representatives of the executive board and the medical director hold guest lectures to present information on the structure and responsibilities of the executive and supervisory boards of the University Hospital in Munich.

Students learn about the political and healthcare systems in Germany in problem-based learning tutorials. Cases covering relevant economic and political issues were designed for use during the tutorials. To conduct the tutorials, SiGma alumni from previous years were given tutor training and, together with the seminar instructors, deployed as tutors.

#### Phase II: Gaming simulation and individual project

The learning objectives for this phase include fostering experience-based, self-directed and problem-based learning about healthcare management and the practical application of the knowledge acquired in phase I. To accomplish this, simulated gaming is used. In a realistic simulation of a supervisory board meeting, students assume the roles of executive board and supervisory board members. Student preferences are respected to the extent possible when assigning roles.

During the preparatory phase, students interview the decision-makers they will be simulating to gather information on points of view and particular interests. To achieve a balance between the levels in the classification described by Cecchini, students are assigned an individual project on a case study, in addition to the role playing and simulation [18]. To enhance the authenticity, this involves a project that has been recently implemented by the University Hospital.

**Figure 2:**
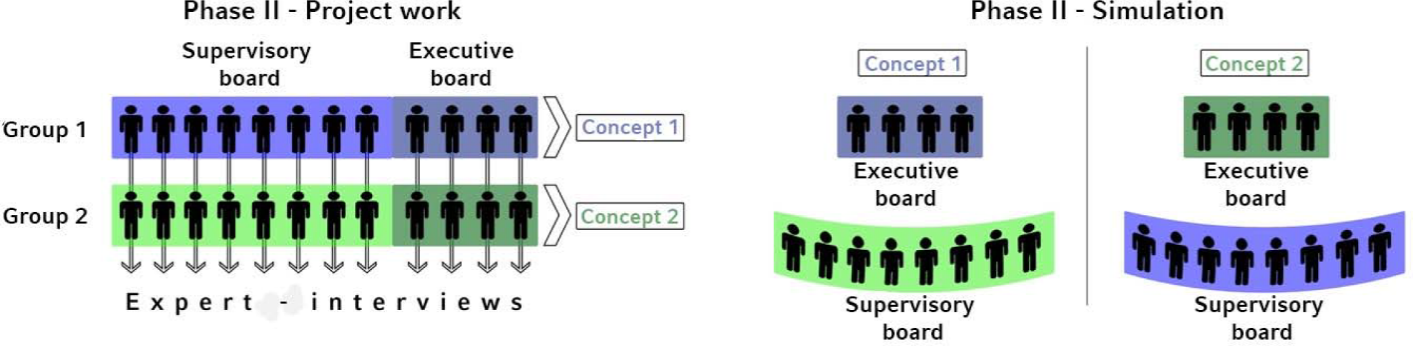
Simulation and Project work in Phase 2

A competitive aspect is created by forming two groups which work separately on the same project and then present their work to each other in simulated board meetings; this turns the simulation into a gaming situation (see figure 2 (Fig. 2)). The board meeting takes place in a conference room at the University Hospital. When students experience this realistic meeting setting, many aspects that are otherwise difficult to impart about decision making processes at the upper management level, such as preliminary agreements, group dynamics and personal relationships, implicitly arise and become visible.

Each board meeting is either tape-recorded or video-recorded by the instructor to give the students comprehensive feedback in the next phase and during the debriefing session.

#### Phase III: Individual project

In this phase the focus is now on establishing and applying the learning objectives from the first two phases, along with the procedural learning objectives (see table 1 (Tab. 1)).

The students work together and separately on a new project that has been selected in advance by the executive board and is on the agenda for discussion at the next (real) supervisory board meeting. The topics covered include human resource management, process management, controlling and reporting, and other project-specific topics (see table 2 (Tab. 2)). During this phase students meet with experts in the particular areas (e.g. medical controlling) and receive any necessary special instruction (e.g. software). At the end of this phase the students present their projects to the executive and supervisory boards who in the past have implemented a number of the resulting ideas and proposals (see table 2 (Tab. 2) and Results).

#### Workshops

Over the course of the semester, a series of workshops are organized for the seminar participants in cooperation with various campus-based and non-academic partners (see table 3 (Tab. 3)). These workshops primarily cover procedural learning objectives such as successful teamwork, effective project management and target-oriented negotiations.

#### Evaluation

The project is evaluated extensively in each of the three phases. The evaluation is based on standardized questionnaires [Likert scale (1=agree completely; 6=disagree completely) and open-ended questions] and structured, spoken feedback rounds at the end of each phase. At the same time, feedback regarding the quality of the projects is solicited from the decision-makers at the University Hospital.

## Results

### Demographic profiles of the attendees

MeCuM-SiGma was held for the fifth time during the 2014/15 winter semester. In the previous four years (2010-14) an average of 22 students (88 total) have attended each course. The mean age for all students was 23.6 years (mean)±2.9 (standard deviation), with no significant deviations between the cohorts (table 4). A total of 62.5% of all participants were male, 37.5% female. Here, differences are visible between the cohorts (table 4). Approximately two-thirds of the participants (63.6%) were in the clinical phase of medical study at the time they took the seminar, around a quarter (27.1%) in the preclinical phase, and the rest were in the fifth and final year of medical study (see table 4 (Tab. 4)). One student who completed the seminar was not a medical student.

#### Evaluation data

The evaluation data collected to date from the four previous cohorts (response rate: 82-100%, depending on cohort) show that, overall, the seminar is rated very positively by students [overall rating on a six-point grading scale (1=excellent; 6=unsatisfactory): 1.28 (mean)±0.45 (standard deviation)]. Students attribute a substantial gain in knowledge to the seminar (1.36±0.62). In addition, they view the information learned as important for their future medical career (1.27±0.51). Students evaluate their understanding of the German healthcare system and the principles of healthcare management as being better after completing the seminar (1.42±0.60 and 1.91±0.84, respectively). Based on the acquired theoretical knowledge and practical skills, the students now feel that they approach projects and assignments with more confidence (2.17±1.25).

In their open-ended responses, students particularly mentioned the relevance of the topics (“The content of MeCuM-SiGma is extremely relevant for every medical student! The concept of SiGma should be expanded!”) and the practical focus of the course (“What I learned is highly relevant to practice”). However, several students were unsure how to actually apply the knowledge gained (“I don’t yet know if I am able to better apply what I learned”).

At the end of each course, all of the cooperative partners were informed by email of the evaluation results and asked for their evaluations of the most recent SiGma cohort. Those at the University Hospital evaluated the collaboration with the students as very positive and beneficial. Those in key positions at the government ministries represented on the supervisory board repeatedly expressed their agreement with and support for the course concept. Furthermore, those serving as role models in the decision-making bodies concretely express their support for this course in that they are willing each year to meet with the participating students for the role interviews.

## Discussion

Despite the fact this seminar is an elective, the application process is quite extensive with an essay and statement of intent, and seminar attendance brings with it a substantial time commitment for students, the excellent evaluations and the constant enrollment numbers demonstrate the positive resonance among the participating students. Based on their responses to the self-assessment questions, students who have completed the seminar also appear to better understand the skills associated with the manager role, as defined in this context, and are possibly in a better position to apply them in their later practice of medicine. Contact with the role models, the close proximity of the project to real situations, and the practical focus have special significance for students according to statements made by participants. The repeated involvement of the participants in actual and important management projects at the hospital shows (see table 1 (Tab. 1)) that the hospital profits in equal measure. At the same time, many innovative ideas and impetus coming from students have been adopted and implemented by the hospital administration within the scope of different projects. This can be viewed as an indirect confirmation of success. As a result of this unique collaboration between the purely practical management structures and university teaching, a mutually beneficial relationship has been established and maintained. Despite the positive evaluations, in-depth analysis concerning the teaching of management skills during medical education is difficult due to a lack of validated assessment instruments [12]. Moreover, additional longer-term studies are needed to investigate transferability and the actual application in day-to-day hospital work.

Most existing programs that focus on training for the manager roll target assistant physicians and originate from North America [12]. Only a few of these programs are oriented directly toward medical students [20], [21], [22], [23]. The structure and learning objectives of individual programs are often very heterogeneous since, as already mentioned, the role of the physician as manager is not yet clearly defined in a sufficient manner. Despite this, many programs follow the CanMEDs and ACGME roles, as does MeCuM-SiGma [24], [25], [26], [27], [28], [29], [30].

Many programs for assistant physicians focus on imparting basic business principles, financing, and leadership skills within the scope of specific specialties [24], [25], [26], [31]. In contrast, the focus of MeCuM-SiGma is broader, placing importance on basic procedural management skills and a general understanding of the healthcare system [20], [21], [23].

A number of established programs use workshops [20], [22], [24], [25], [32], [33] and lectures with discussions [20], [21], [22] as teaching methods. Similar to MeCuM-SiGma, one program uses interactive seminars lead by co-workers or student peers [34][. Another parallel to this program is the assignment to work on a management project, however, this is designed to be a group assignment, rather than an individual one. MeCuM-SiGma distinguishes itself from these other programs through its combination of the various methods in gaming simulation. This approach is being increasingly used in business studies curricula [35]; we are unaware of any such course in medical education that offers preparation for the manager role in medicine.

The diversity of the teaching methods and the participation of a large number of university-based and non-academic partners make course organization very extensive, but worthwhile. For this reason the course can only be offered to a limited number of motivated students. In our opinion, the concept behind MeCuM-SiGma can also be successfully applied at other medical schools or institutions. The crucial factors, in our experience, are close cooperation with the decision-making bodies when selecting and conducting projects and the constant and close supervision of the students during the project phases.

## Conclusions

Both the results from various research papers and the initial data from the evaluations demonstrate the need among medical students for better instruction and training regarding the manager role. The seminar, MeCuM-SiGma, attempts to fill this gap in the medical curriculum at the two universities in Munich, Germany.

The positive evaluation data and feedback from collaborating partners suggest our efforts have been successful. Still, it will be important in the future to define the role of the physician as manager more clearly to focus the education of physicians-to-be even more on the challenges facing those in medicine. Further research is needed on the necessary medical management skills. Integration into the core curriculum is also imperative. When implementing this, a tighter interlinking with the interdisciplinary subject area of medical economics, healthcare systems, and public health is desirable, for instance in the form of joint seminars and course planning.

## Competing interests

The authors declare that they have no competing interests.

## Figures and Tables

**Table 1:**
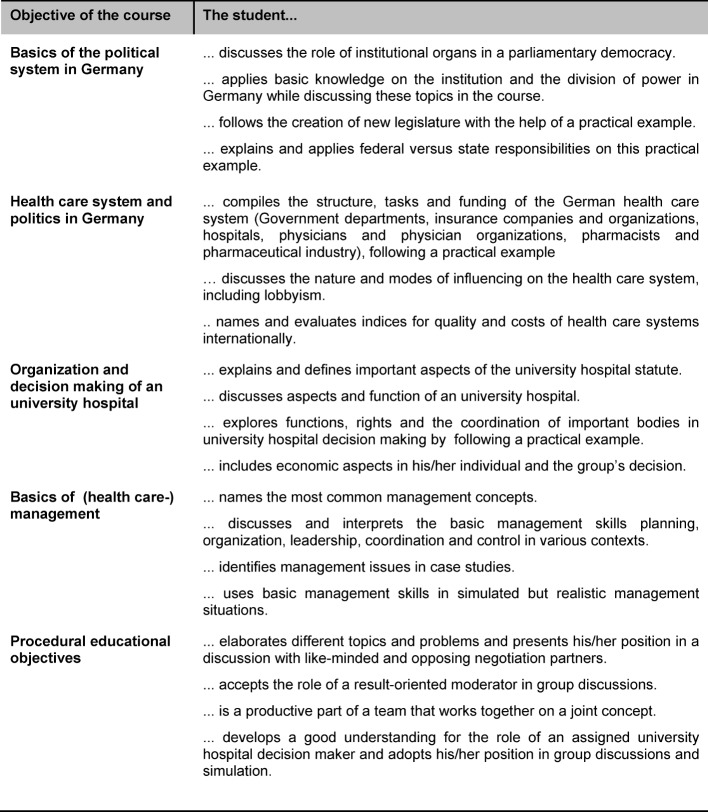
Educational objectives

**Table 2:**
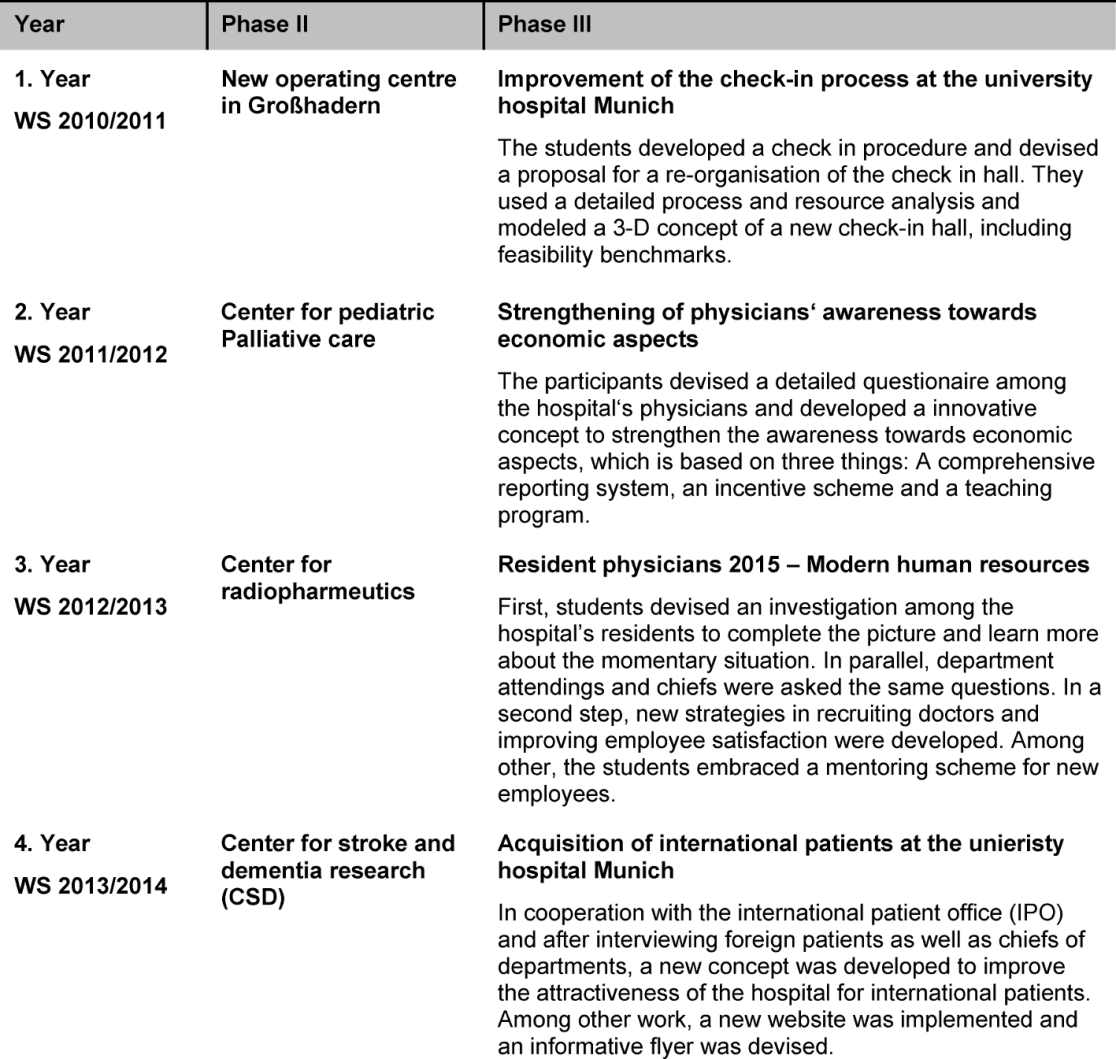
Overview of the projects in Phase 2 and 3

**Table 3:**
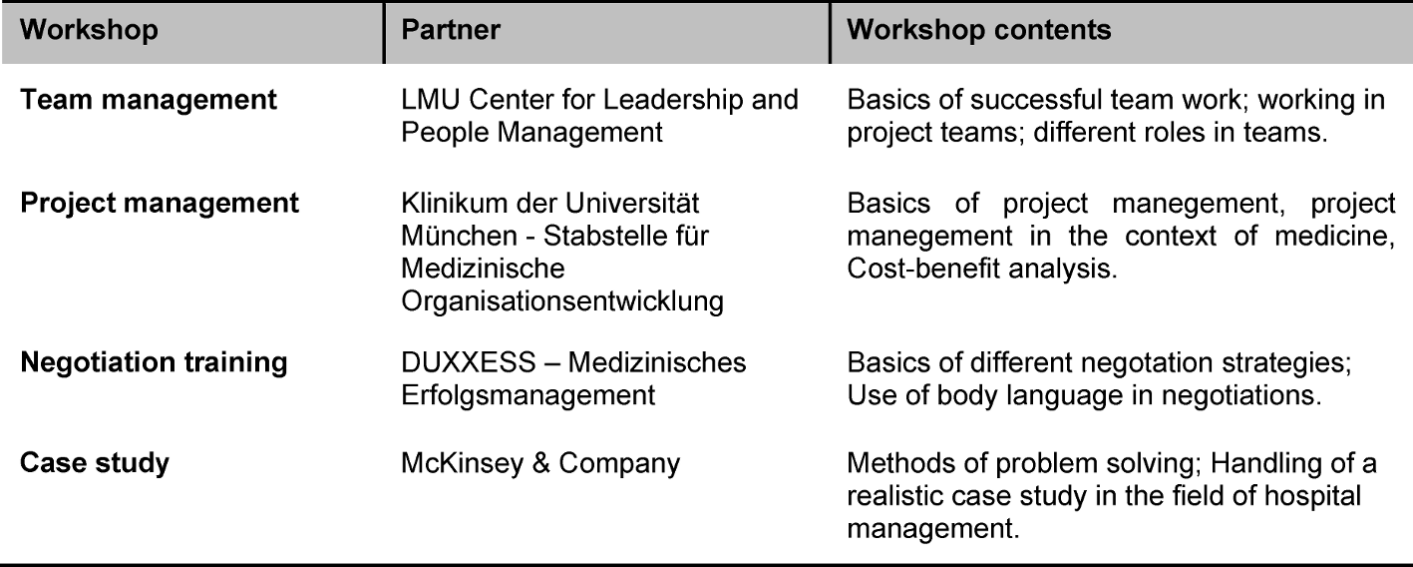
Overview over concomitant workshops

**Table 4:**
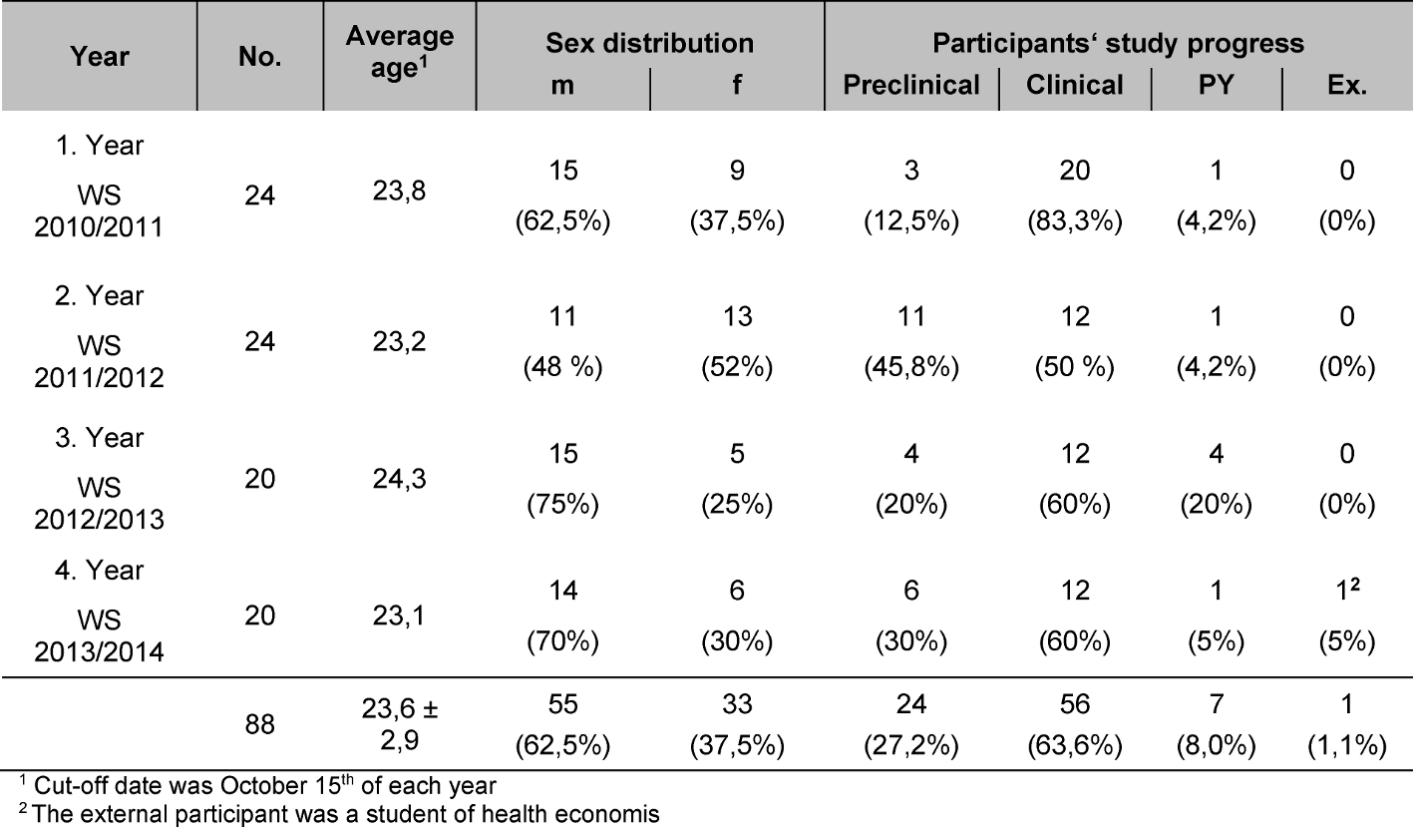
Tabelle 4: Demographische Daten der Teilnehmer
